# The art and science of using quality control to understand and improve fMRI data

**DOI:** 10.3389/fnins.2023.1100544

**Published:** 2023-04-06

**Authors:** Joshua B. Teves, Javier Gonzalez-Castillo, Micah Holness, Megan Spurney, Peter A. Bandettini, Daniel A. Handwerker

**Affiliations:** ^1^Section on Functional Imaging Methods, Laboratory of Brain and Cognition, National Institute of Mental Health, National Institutes of Health, Bethesda, MD, United States; ^2^Functional MRI Core Facility, National Institute of Mental Health, National Institutes of Health, Bethesda, MD, United States

**Keywords:** fMRI, quality control, neuroimaging, reproducibility, resting state, GLM, noise removal

## Abstract

Designing and executing a good quality control (QC) process is vital to robust and reproducible science and is often taught through hands on training. As FMRI research trends toward studies with larger sample sizes and highly automated processing pipelines, the people who analyze data are often distinct from those who collect and preprocess the data. While there are good reasons for this trend, it also means that important information about how data were acquired, and their quality, may be missed by those working at later stages of these workflows. Similarly, an abundance of publicly available datasets, where people (not always correctly) assume others already validated data quality, makes it easier for trainees to advance in the field without learning how to identify problematic data. This manuscript is designed as an introduction for researchers who are already familiar with fMRI, but who did not get hands on QC training or who want to think more deeply about QC. This could be someone who has analyzed fMRI data but is planning to personally acquire data for the first time, or someone who regularly uses openly shared data and wants to learn how to better assess data quality. We describe why good QC processes are important, explain key priorities and steps for fMRI QC, and as part of the FMRI Open QC Project, we demonstrate some of these steps by using AFNI software and AFNI’s QC reports on an openly shared dataset. A good QC process is context dependent and should address whether data have the potential to answer a scientific question, whether any variation in the data has the potential to skew or hide key results, and whether any problems can potentially be addressed through changes in acquisition or data processing. Automated metrics are essential and can often highlight a possible problem, but human interpretation at every stage of a study is vital for understanding causes and potential solutions.

## 1. Introduction

The fundamental question that a quality control (QC) process should answer is, “Will these data have the potential to accurately and effectively answer my scientific question and future questions others might ask with these data?” The secondary goal of QC is to identify data anomalies or unexpected variations that might skew or hide key results so that this variation can either be reduced through data processing or excluded. Even for a perfectly designed study, problems can arise during nearly every step of the data acquisition and analysis. While a specific problem might be unexpected, the existence of problems should be expected. Failure to check the quality of data will result in incorrect or misleading interpretations of data. Therefore, a QC process should be a fundamental element in the design of any study. While good QC processes will not guarantee good results, they can greatly reduce the chances of generating misleading or incorrect results.

QC is both a key part of scientific progress in fMRI and a neglected topic. Overviews of good practices mention the importance of a good QC process (Poldrack et al., 2008; Nichols et al., 2017), but do not describe the elements of a good QC process in depth. Detailed QC protocols for fMRI studies tend to be published only for large or multi-site studies, do not always present context, and only a few include operating procedures for non-automated steps (Friedman and Glover, 2006; Marcus et al., 2013; Alfaro-Almagro et al., 2018; Kim et al., 2019; Scott et al., 2020; Huber et al., 2021; Huguet et al., 2021). Publications and seminars that systematically discuss and debate expectations and methods of QC for fMRI are rare. Automated or semi-automated QC tools have long been part of fMRI processing pipelines (Cox, 1996) and there is a growth in QC tools for specific phases of acquisition and processing (Dosenbach et al., 2017; Esteban et al., 2017; Heunis et al., 2020). Still, despite the central importance of good quality data for scientific reproducibility, there is only a modest amount of education and methods development research that focuses on improving QC processes.

Our anecdotal experience is that learning how to think about fMRI QC and the practical parts of checking data are often taught through hands-on training, particularly when people acquire data. With a rising number of researchers working with shared data and not acquiring data, a smaller proportion of neuroimagers may be receiving this necessary training during formative career stages. This is paired with an assumption that data that are published and shared are reasonable quality data. We have repeatedly heard shared datasets being referred to with terms such as “gold standard data,” which is another way of saying data users think they can trust downloaded data without running their own QC process.

To reduce these training gaps and push for more work and innovation, we document our approach to fMRI QC with two goals in mind: (1) Outline a quality control framework for fMRI for scientists who have not learned these skills during formative training periods. (2) Highlight QC priorities for a researcher who uses data they did not collect. We demonstrate a QC process, primarily using AFNI software, on a sample dataset as part of the FMRI Open QC Project.^1^ For this project, multiple groups demonstrate their QC procedures with a variety of software packages on the same data.

While no manuscript can replace hands-on training, we highlight ways of thinking about fMRI QC that may guide additional learning. Our framework and demonstration are centered on the idea that automation should augment rather than replace human judgement. Also, discussions about QC often focus on what data to accept vs. exclude, but timely human judgement can identify problems that can be corrected through changes in acquisition and analysis. This interaction between automation and human judgement will become more critical to understand and improve as fMRI datasets increase in size. Large studies require a clear plan for which aspects of QC can be automated and where the finite amount of human intervention and judgement is most useful. To that end, we provide a framework for thinking about general approaches with a specific focus on where human intervention is particularly important.

## 2. Quality control framework for fMRI

QC asks whether and how data can be used. For fMRI data, this comes down to addressing two questions (1) Which voxels have useable data? (2) Are the locations of those voxels in the brain accurately defined? Answers to the first question involve ensuring consistent fields of view across all scans, computing basic QC metrics such as signal-to-noise ratio (SNR) and the temporal-signal-to-noise ratio (TSNR), and searching for spatial and temporal artifacts which may render these areas unreliable for modeling. Answers to the second question involve looking at functional alignment between runs, functional to anatomical alignment, anatomical alignments to a common stereotaxic space, and anatomical alignments across study participants.

The quality checks needed to answer these questions are not the same for all study purposes and the best tools to answer them vary by study phase and purpose. As discussed in a generalized QC framework by (Wang and Strong, 1996), QC includes both intrinsic and contextual measures. Intrinsic measures characterize inherent properties of the data. For example, the average temporal-signal-to-noise ratio (TSNR) of gray matter voxels might be intrinsically useful. However, contextual measures depend upon the research hypothesis. For example, the TSNR values of voxels in the temporal pole might only matter in the context of studies with hypotheses about the temporal pole. Similarly, some functional-to-anatomical alignments are intrinsically poor, but an imperfect alignment might be sufficient in the context of a study that focuses on large regions-of-interest (ROIs) or spatially smoothed data. As another example, a modest amount of head motion or breathing artifacts might be addressable through data processing for some studies but could be problematic in the context of a study with task-correlated breathing (Birn et al., 2009) or with population biases in head motion (Power et al., 2012). This distinction between intrinsic and contextual quality is critical because many discussions of fMRI QC focus on whether to keep or exclude data, yet there are often situations where data can be processed to be useful for a subset of potential applications, underscoring the need to keep the application of data central when assessing quality.

We organize our QC framework into four phases: during study planning, during data acquisition, soon after acquisition, and during processing. This structure should guide when to think about certain steps, but the same overall issues cross all phases, and they are not in a strict temporal order. For example, an issue identified during processing may prompt changes to study design or acquisition. An additional element of QC is QC of the acquisition hardware, which should be checked regularly as part of the operational procedures of any fMRI research facility. Since there are already multiple resources for this type of fMRI QC (Friedman et al., 2006; Liu et al., 2015; Cheng and Halchenko, 2020), we are limiting our scope to QC that is specific to the data collected during a study. The appendix summarizes the suggestions in this framework for use as a guide when designing a study-specific QC protocol.

### 2.1. QC during study planning

Good QC procedures depend on having the QC-relevant information stored in a representationally consistent manner where they can be efficiently accessed (Wang and Strong, 1996). This requires effort during the planning stage of a study to make sure this information will be identified, collected, and organized. Defining QC priorities during the planning phase also supports future data sharing. The information that needs to be organized to support a robust QC protocol will also be accessible to future users of the data.

Expert study-specific advice is highly recommended during study planning. If one has access to experts in experimental design and acquisition, seek out their advice during this phase rather than the “What is wrong with my data?” phase. Many of the QC protocols referenced in the introduction feature study-specific examples and show how others have prioritized and organized QC-relevant information. Key topics to consider when planning a study are:

- What QC measures will support the goals of the study? For example, if a study has *a priori* ROIs then QC measures for those ROIs and pilot scans that optimize those QC measures can flag issues that prompt acquisition changes and avoid wasted data.
- Minimize variability in operating procedures across scan sessions by generating checklists and written instructions that clearly describe what experimenters should do during the scan (e.g., acquisition instructions), and should tell to participants [e.g., clear task or rest instructions and protocols to decrease head motion (Greene et al., 2018)]. The same applies to preprocessing and QC measures to calculate soon after each scan so that issues can be efficiently identified. (Strand, 2023) is a general overview for how good procedures can help avoid errors and improve data quality.
- What data should be collected during acquisition that will support QC later? This includes both logs of expected and unexpected events such as: participant behavior (e.g., task behavioral response logs, feedback from participants, observed movement during runs, seemed to fall asleep in a run, needed to leave scanner & get back in), issues with stimulus presentation, qualitative observations and quantitative measures of real-time data quality, respiratory and cardiac traces, external sources of variation between participants [e.g., time of day, caffeine intake, endogenous and exogenous sex hormone variation (Taylor et al., 2020)] and all scanning parameters.
- How QC measures will be organized and shared. Acquisition-stage QC is useful only if it is connected to the data, understandable by others, and easy to share.
- Finally, pilot sessions should go beyond attempting to optimize MRI acquisition parameters, to play a role in addressing all the above QC topics, so that when acquisition for a study begins, the procedures for acquiring, organizing, and rapidly checking QC metrics are already in place.

### 2.2. QC during data acquisition

It is better to design and follow a QC-focused scanning protocol and proactively collect good data than to retrospectively attempt to remove or fix bad data. That means one should aim to look at reconstructed MRI data as soon as feasible to identify unusual dropout or serious artifacts. When scanners are equipped with real-time fMRI capabilities, this initial inspection can happen as volumes are being acquired. While all modern scanners allow people to look at volumes during a scanner session, additional, real-time systems such as AFNI (Cox and Jesmanowicz, 1999) and NOUS (Dosenbach et al., 2017) can help identify artifacts in time series and excessive motion events, prompting researchers to notify the participant and to re-collect data. Real-time quality checks should be extended to any concurrent peripheral measurements such as respiratory or cardiac traces, behavioral responses, EEG, and eye tracking, to name a few. Stimulus presentation scripts can also integrate some rapid feedback so that experimenters can identify participants who are not performing a task as expected. Even if a session-specific issue observed during acquisition is not correctable in real-time, it can be flagged during acquisition for closer attention during processing or can lead to protocol changes to improve future scanning sessions.

### 2.3. QC soon after acquisition or download

Rapid QC after acquisition can focus on intrinsic issues that might not have been obvious during acquisition. If done between acquisition sessions, information gathered this way can identify ways to improve future acquisitions and avoid unexpected downstream analysis problems. The most important thing to check is that the expected data are present, have understandable and accurate file names, and are properly documented. Shared datasets often have a few surprises (e.g., missing or corrupted files, duplicated data, incomplete runs). For example, early QC can help identify and fix a task presentation script that insufficiently logged behavioral responses and times. These early checks should also include confirming that each MRI run and peripheral measurement, such as respiration and cardiac traces, have the correct number of samples, and look as expected. Checks should also determine if fMRI data look anatomically correct and have consistent orientation and brain coverage. This should also include checking whether parameters in data headers are plausible and match documentation. For example, we recently saw a dataset where the publication accurately listed a slow 5.1 s TR for a specialized sequence, but the files were incorrectly saved with a 1.5 s TR in their headers. This caused problems when processing steps read the incorrect TR from the file headers.

For shared data, check if there is any information about the QC procedure or a list of excluded runs or participants. If there is no information on problems with the data, that is likely a warning sign that there was no systematic QC procedure, and one should examine the data more carefully before using. If there was a clear QC procedure, one can also check if contextual metrics for newly planned analyses were included. For example, if the initial analysis focused on task responses and new plans focus on connectivity measures, the initial QC may not have focused on potential temporally correlated artifacts.

While full processing of data can be a slow process, an initial, limited preprocessing aimed at generating key automated QC metrics should be run as soon as possible. Even if a full preprocessing pipeline is not finalized, running some basic preprocessing steps can identify issues that will help tally what data are useable and can help better optimize the final preprocessing pipeline. For example, if anatomical to functional alignment is poor in many participants during initial preprocessing, then time can be devoted to figuring out ways to optimize the alignment algorithms for a given dataset.

### 2.4. QC during data processing

The big advantage of integrating QC into a data processing pipeline is that QC metrics and key images for visual inspection can be automatically calculated for multiple steps in the pipeline. For example, AFNI’s afni_proc.py pipeline automatically generates a QC html page with values and images that aid human interpretation of data quality. By compiling automatically calculated measures, someone with modest training can view reports to identify many things that look odd and are worth showing to a more experienced researcher.

While the processing steps have a fixed order, examination and interpretation of QC measures do not. Therefore, automated QC pipelines should calculate and organize measures from across the processing stream to aid human interpretation. This is particularly true for shared data where issues with unprocessed data may not have been checked or documented. For example, a few authors were recently working with a shared dataset where the acquired slices did not cover the most superior 5 mm of the cortex. This was flagged as a failure of the registration algorithm, but by going back to the unprocessed data, it became clear that the alignment was fine, but data were missing.

After data are processed, check if there are any warnings or errors from the execution of the processing script. These may seem obvious, but subtle downstream errors from unnoticed script failures happen. This is also the easiest place to see if the same warnings repeatedly appear and warrant changes to a processing pipeline. AFNI makes this easy by compiling the warnings from all processing steps in AFNI’s QC output so that users can look in one place to see if any parts of the script failed to execute or if serious data issues were automatically flagged.

Then quality checks can be separated into answering the two questions from the beginning of this section: (1) Which voxels in a dataset have usable data? (2) Are the locations of those voxels in the brain accurately defined?

#### 2.4.1. QC during data processing: Usable voxels

The most straightforward check is noting areas of the brain that were included in the scan’s field-of-view. Since most pipelines attempt to mask out non-brain voxels, one must make sure the mask is not excluding brain voxels or retaining voxels outside the brain. fMRI data always suffers from signal dropout and distortions, so voxels within the brain are expected to be missing, but, for a study with the same acquisition parameters, the location and amount of dropout and distortion should be relatively consistent. A dataset with unusually large amounts of dropout should be checked to see if there are other issues. Even if dropout is fairly consistent, the QC process should identify voxels with usable data in only a subset of participants. Particularly for ROI-based analyses and connectivity measures, voxels with data in only a fraction of a population can cause non-trivial biases in data that are hidden under ROI averages or averaged group maps.

The temporal signal-to-noise ratio (TSNR = detrended mean/standard deviation) is a rough, but useful measure of fMRI quality that highlights issues that can be missed by looking only at the magnitudes, since the standard deviation of time series will be affected by temporal acquisition artifacts and head motion spikes. On a voxel-wise map, the spatial pattern of TSNR values can vary based on acquisition options. For example, a 64-channel head coil with many small receiver coils will likely have relatively higher TSNR values on the surface versus the middle of the brain compared to a 16-channel coil (although the raw TSNR values should be higher everywhere). In addition to viewing TSNR maps, with consistent acquisition parameters, TSNR should be similar across a study, so data warrants closer examination if the average TSNR for the whole brain, white matter, or gray matter is lower in some runs.

Mean images and TSNR are useful for identifying potential problems, but not necessary for understanding causes and potential solutions. By recognizing different types of MRI artifacts, it is possible to figure out if a problem can be solved through data processing, or censoring time points or voxels. Not every artifact is a problem. For example, the differences in TSNR between the surface & the center of the brain with multi-channel head coils is not inherently a problem, but it can affect studies that directly compare or correlate cortical surface and subcortical responses (Caparelli et al., 2019). MRI imaging artifacts are best understood with hands-on training, but there are some key things to look for. Any contrast changes that do not seem to follow brain tissue or are not symmetric between hemispheres might be artifacts. It is important to look at data from multiple views (i.e., axial and sagittal) because some artifacts may be obvious within acquired slices and others may be visible across slices. If there is a bright artifact in one location, it might be possible to exclude data from that location, but many types of artifacts are obvious in one location and present, but less obvious over a larger portion of the brain, which would make data unusable. Processing that includes masking or temporal scaling of the data can often hide these artifacts, but they can be more visible in TSNR versus mean images or if the contrast is adjusted to give values nearer to zero more brightness. Another useful tool is to look at power spectra of data, which can identify if an artifact is fluctuating at consistent frequencies. Temporally periodic artifacts can be due to acquisition problems that might affect an entire dataset or by respiratory and cardiac fluctuations which are potentially addressable.

If the brain volume overlaps itself or there is a replicated part of the brain where it should not be, this wrapping or ghosting can inject signal from one part of the brain into another part and make a run unusable. A way to examine the seriousness of a ghosting or wrapping artifact is to correlate the rest of the brain to voxels within the artifact. AFNI’s *instacorr* interface lets users interactively correlate data to specified voxels and is particularly useful for this. *Instacorr* does not depend on AFNI processing so it can be used on data processed with other packages. If a voxel in an artifact is correlated with other clusters of voxels in a non-anatomical pattern (e.g., The signal in one brain region correlates with the same-shaped ghosted region elsewhere in the volume) that is a serious sign that the artifact corrupted the data.

One additional tool for identifying temporal artifacts in voxels is to look at partially-thresholded and unmasked activation maps for both task-locked GLM models and correlations to the global averaged signal or white matter. While one cannot reject a dataset if the task of interest is not significant, if a study uses a visual task and there is no task-locked activity in the primary visual cortex, then there are likely additional issues with the data. If there is task-locked activity outside of the brain or on tissue/CSF boundaries, that is a sign of ghosting, motion artifacts, or task-locked breathing (Birn et al., 2009). If there is not a task, correlation maps can highlight similar issues, but they can also be used to identify population differences. For example, given the widely documented differences in global signal across populations (Power et al., 2012; Gotts et al., 2013; Yang et al., 2014), any study that plans to regress out the global signal as noise needs to correlate the global signal to the other voxels in the brain and test whether the correlation between the global signal and voxels systematically varies between populations or other contrasts of interest.

There are many automated QC metrics, in addition to TSNR, that can be used to automatically exclude data in voxels or highlight areas of concern. The most common ones are spike detection and motion estimates. Those can be used to both censor specific volumes and to automatically decide whether a run has too many censored volumes to be useable. The remaining degrees of freedom (DOF) after temporal filtering, censoring, and noise regression can be used to decide if sufficient DOF remain for statistical tests. The effect of temporal filtering on the loss of degrees of freedom is sometimes ignored in fMRI studies. AFNI also outputs a spatial smoothness estimate for each dataset. These numbers are not especially useful in a single run, but for a given set of acquisition parameters, the smoothness estimate should be roughly consistent across a study. If smoothness estimates vary widely, it is worth looking more carefully at outlier runs.

#### 2.4.2. QC during data processing: Alignment

Evaluating individual voxel data quality benefits greatly from automation, but masking and alignment results often require manual inspection and interpretation. This is because different acquisitions can have different contrasts and parameters, so what works well for one dataset might not work as well for another. Artifacts and non-trivial spatial distortions in unprocessed data can also affect masking and alignment. Automated metrics for alignment quality will keep improving, such as with a metric to automatically warn that the left and right sides of the brain are flipped (Glen et al., 2020). Automation can be used to compile images that facilitate human inspection. AFNI’s html reports include images where the sulcal edges from a participant’s anatomical volume are overlayed onto the functional images or common anatomical templates. This is a quick way to catch clearly mis-aligned brain edges or sulci and potential issues that are worth a closer examination of the full volumes’ alignments.

Visual checks can focus on several factors. If collected during the same session, an anatomical image should have a decent alignment to the functional data even without processing. Atypical brain structures can be viewed before processing. An expert can tell which types of variation are concerning – either to the volunteer or to data processing – but a less experienced reviewer can flag anything that is asymmetric for expert review. Benign cysts, larger ventricles, and other atypical structures do not require rejecting data, but they can affect spatial alignment between participants as well as the locations of functional brain areas. As such, those occurrences should be noted, and more attention should be spent on assessing alignment quality.

Since most fMRI research uses multi-channel receiver coils, one very common artifact is intensity inhomogeneity, where the voxels closest to the head coil have a higher magnitude signal than voxels nearer to the center of the brain. This inhomogeneity can look bad, but it is not inherently a problem. That said, it can affect the accuracy of brain masking and alignment so, if the data has a lot of inhomogeneity, it is useful to spend more time checking brain masking and alignment.

It is worth taking time to make sure a brain mask excludes sinuses and non-brain tissue, and that a mask does not remove parts of the brain. Inconsistent masking often leads to flawed anatomical-to-functional alignment and flawed reregistration between participants. Unless problems are caused by artifacts or distortions, it is often possible to fix alignment issues by tuning function parameters or by hand-editing masks.

Once many participants in a study are processed and aligned to a template, a summation of all the fMRI coverage maps is very useful for identifying brain regions that are included in only a portion of study participants. Excessive blurring on the average of the aligned images can also signal faulty alignment for a subset of participants. From our experience, looking at such coverage maps is strangely uncommon. A concatenated time series of all anatomical images and an average anatomical are very useful for checking the consistency of alignment across a population.

### 2.5. Peripheral measures

QC for fMRI studies often focuses on the MRI data, but unprocessed and processed peripheral measurements can also be sources of error. While many peripheral measures can be collected and checked, we will highlight a few examples for how to think about such measures in general. To be used with fMRI, peripheral measures need to log their timing in relation to fMRI volume acquisitions. Errors can arise in peak detection for respiratory and cardiac traces. Movement of a finger within a pulse oximeter can create noisy sections with what looks like rapid changes in heart rate that can negatively affect some peak detection algorithms. Anyone who collects respiratory data will also find spontaneous breath holds, which will affect fMRI data. Breath holds will cause large, brain-wide signal changes that bias results or merely be a non-trivial source of noise. For task-based fMRI, check response logs to confirm the expected information was logged and participants were compliant with task demands. Also check to make sure that head motion or respiration patterns are not task-correlated, since non-neural signal sources that are task-locked will bias results.

For all QC steps, it is crucial to consider that algorithms often fail in subtle ways rather than with clear errors, and these are the hardest errors to catch. It is therefore imperative that all steps be thoroughly vetted to ensure all assumptions required by the program are met and that programs are used consistently with their documented intent.

## 3. Methods

The previous section contains information on how to think about planning QC for a dataset. The following examples on shared datasets show how some of these concepts work in practice. As already noted, a QC process checks both intrinsic quality measures and contextual measures that are often dependent on the scientific question that a researcher has in mind. Additionally, because the data have already been collected, we do not demonstrate the phases of QC before and during data collection (though in the discussion we will note some operational steps that could have been taken with these data). Since, we do not know the intended purposes for these data, we can make some assumptions about context, but our attention will primarily focus on intrinsic QC. We are focusing our contextual QC on issues that might affect connectivity measures for rest data or task responses for task data, without making assumptions about regions of interest.

We classify data which we believe could answer such questions as “included,” data which could not answer common or basic questions as “excluded,” and data which may be suitable for some questions but not others as “unsure.” Automation scripts were used to ensure consistency across subjects; the full processing and figure generation code and instructions may be found in our GitHub repository.^2^ Each processing step was given its own script with the expectation that users could check results before proceeding.

Data were initially checked using a basic visual inspection to identify anything of concern in the data including missing information, artifacts, whether the image field of view included the whole brain (excluding the cerebellum and brain stem), and whether there were noticeable anatomical or image abnormalities. Concerns were noted, and screenshots were uploaded to a shared folder. Anything requiring additional discussion prompted either a message or a video chat between researchers to either (a) decide that the object of concern was inconsequential or (b) properly identify the problem and mark it.

For processing of the data after these inspections, T1 anatomical images were segmented using freesurfer’s *recon-all* (Fischl and Dale, 2000), and a non-linear transformation for warping anatomical images to the MNI template space was calculated using AFNI’s *@SSwarper* (Cox, 1996). *SSwarper’s* output includes QC images, which were checked both to make sure that the brain mask had complete coverage and did not include skull, and that the individual brain had been properly aligned to the MNI template.

AFNI’s *afni_proc.py* program was used to perform slice timing correction, rigid-body motion correction, alignment of anatomical and echo-planar images, blurring to 6 mm full-width half-maximum, and regression of physiological-and motion-related signals. Volumes which contained more than 0.25 mm of head motion from neighboring volumes were censored. Voxels which were determined to be outliers by AFNI’s *3dToutcount* were tallied and volumes which had more than 5% of voxels as outliers were censored.

For all data, the *ANATICOR* method (Jo et al., 2010) was used to compute regressors associated with scanner instabilities and physiological noise. In addition, we also regressed motion estimates and their first derivatives. For rest data (subjects 101–120), additional regressors were used to bandpass between 0.01 and 0.1 Hz, which significantly reduced the remaining DOF for the data. For task data, this step was omitted.

In the case of task data (subjects 001–030), tasks were modeled using the simplified task timings supplied with the data. The labeled task conditions were “control” and “task,” and each trial had an onset time and duration. We modeled task responses in our GLM with AFNI’s default double-gamma hemodynamic response function using both the onset and duration information.

For inspecting the outputs of all other steps, we relied primarily on *afni_proc’s* webpage-based QC report. Many figures in this manuscript use QC images that were automatically compiled in this report. Automatic motion correction and outlier censoring were used to see whether subjects exceeded 20% of volumes censored; in these cases, subjects were excluded.

The echo planar image (EPI) to anatomical alignment was checked by ensuring that anatomical edges matched the gyral shapes on the EPIs, that the ventricles were aligned, and that the brain was not distorted to the point of being displaced past the anatomical boundary.

Anatomical-to-template alignment was checked by ensuring subject-warped edges matched the template image’s edges, and, that the gyral shapes on both the anatomicals and the brain edges matched. The final EPI mask was checked to ensure it covered all likely areas of interest (i.e., those targeted by scientific inquiry).

Model fits for regressors of interest were examined to make sure that good fits were not spatially aligned with previously identified artifacts. A similar inspection was performed for seed-based correlation maps to make sure that the underlying correlation structure was free of artifactual patterns.

For the task data, while we do not know the expected patterns, the modeled task responses were examined to see if they presented a plausible design with a sufficient number of uncensored trials per task condition.

Lastly, the warnings automatically generated by *afni_proc* were checked: these include unusually high correlations with nuisance regressors, total percentage of censored volumes, pre-steady-state detection, possible left–right flips, and EPI variance line warnings. For likely left–right flips, without additional information, we cannot ascertain whether the EPI or anatomical has the correct orientation; thus, such subjects are marked for exclusion. EPI variance line warnings are a marker of potential temporal artifact and *instacorr* was used to examine potential artifacts for severity.

## 4. Results

**Figure 1:**
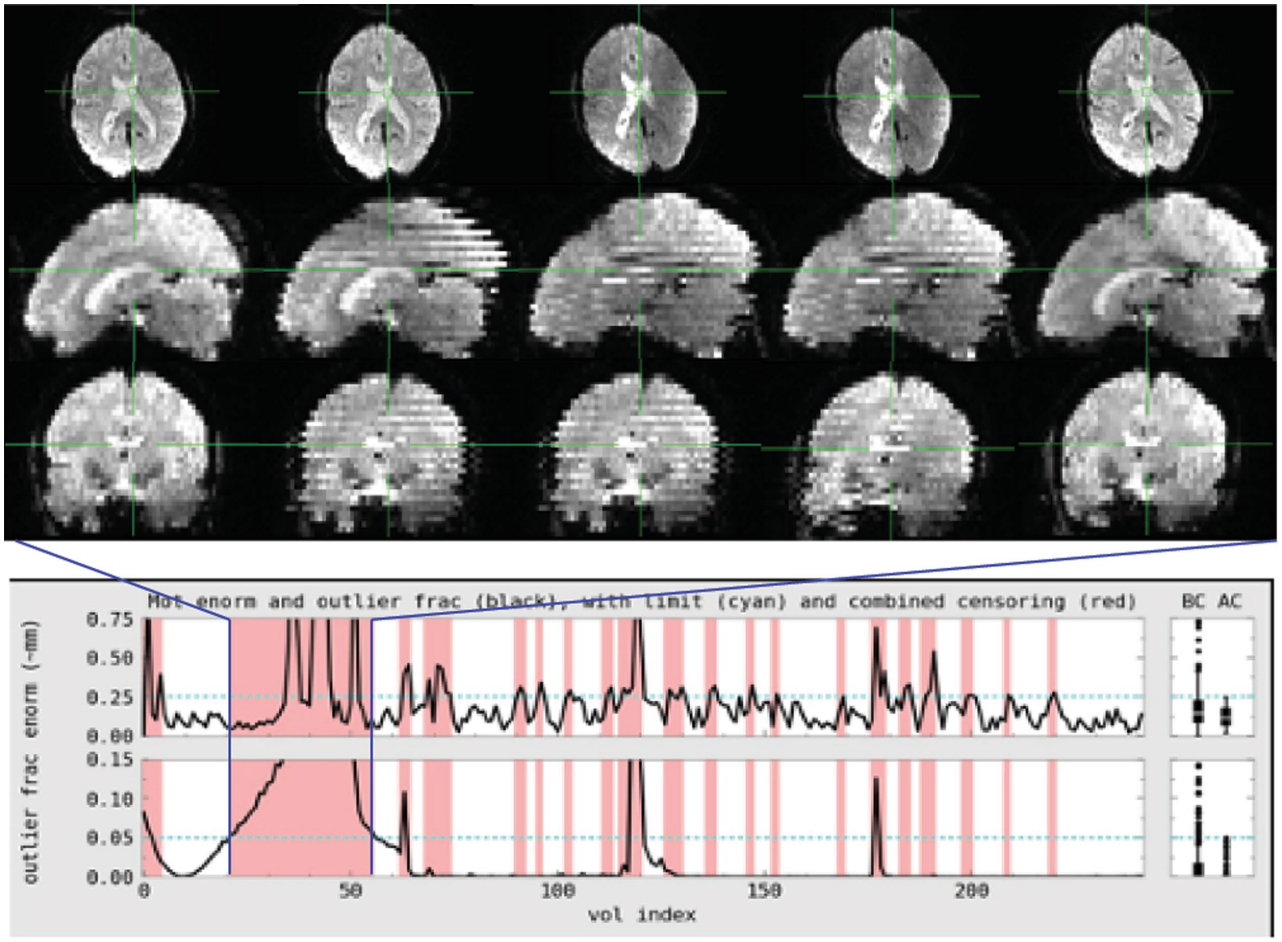
Subject 017 had a high number of censored volumes due to motion. This figure depicts several volumes in which the motion artifact is very clear. Banding due to the magnitude of head motion during acquisition are visible on the sagittal and coronal slices. Within the axial slice, this motion makes part of the lateral ventricles disappear because of displacement during acquisition. Such a large motion artifact should be visible on the console even in an axial-only view. Operationally, it would be useful to note this during acquisition and consider collecting an additional run while the subject is present.

**Figure 2:**
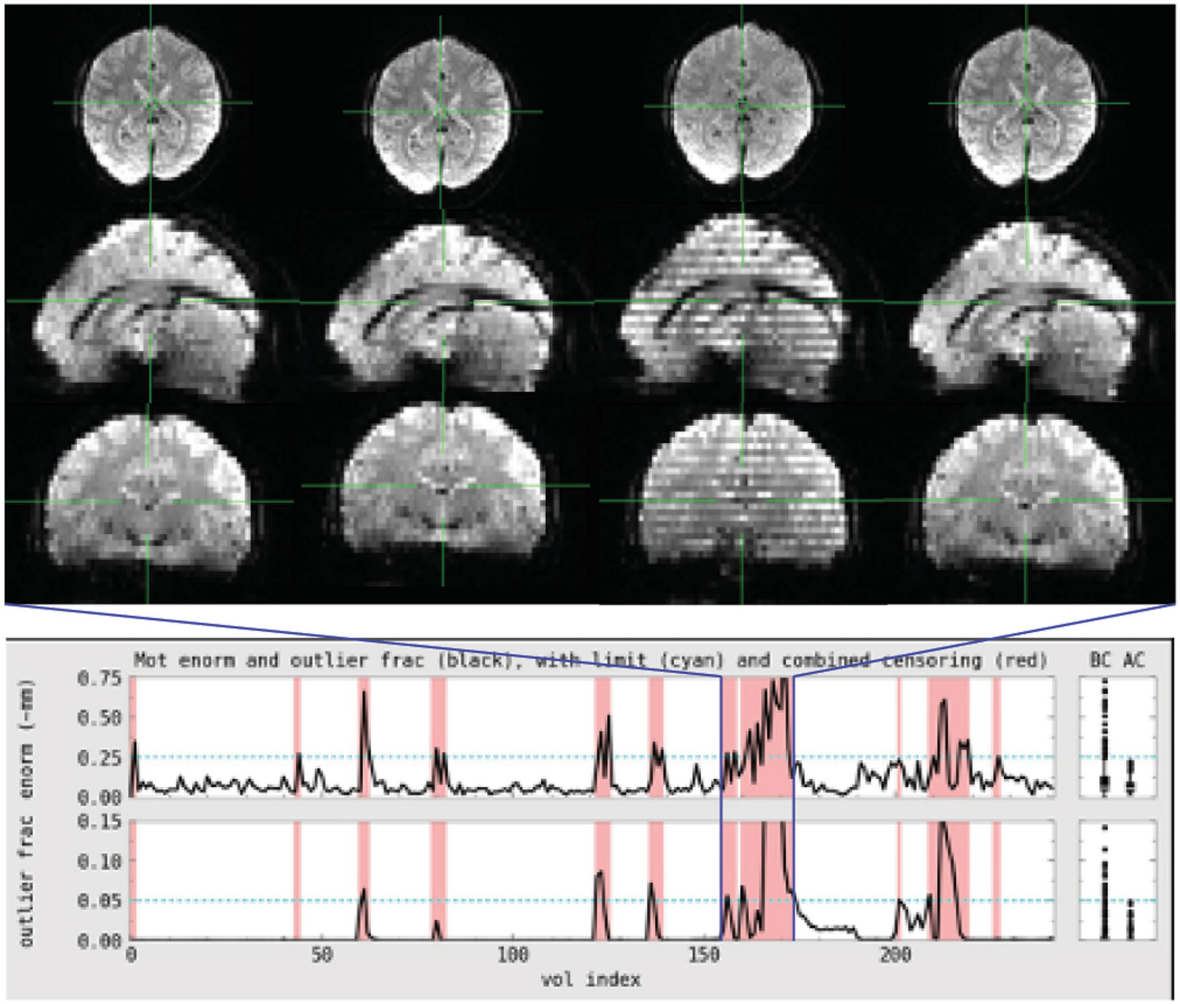
Subject 029 had a more subtle motion artifact than depicted for subject 017. The banding is visible during the period with the most motion but is otherwise more subtle and would be less likely to be noticed during acquisition without automated QC metrics.

**Figure 3:**
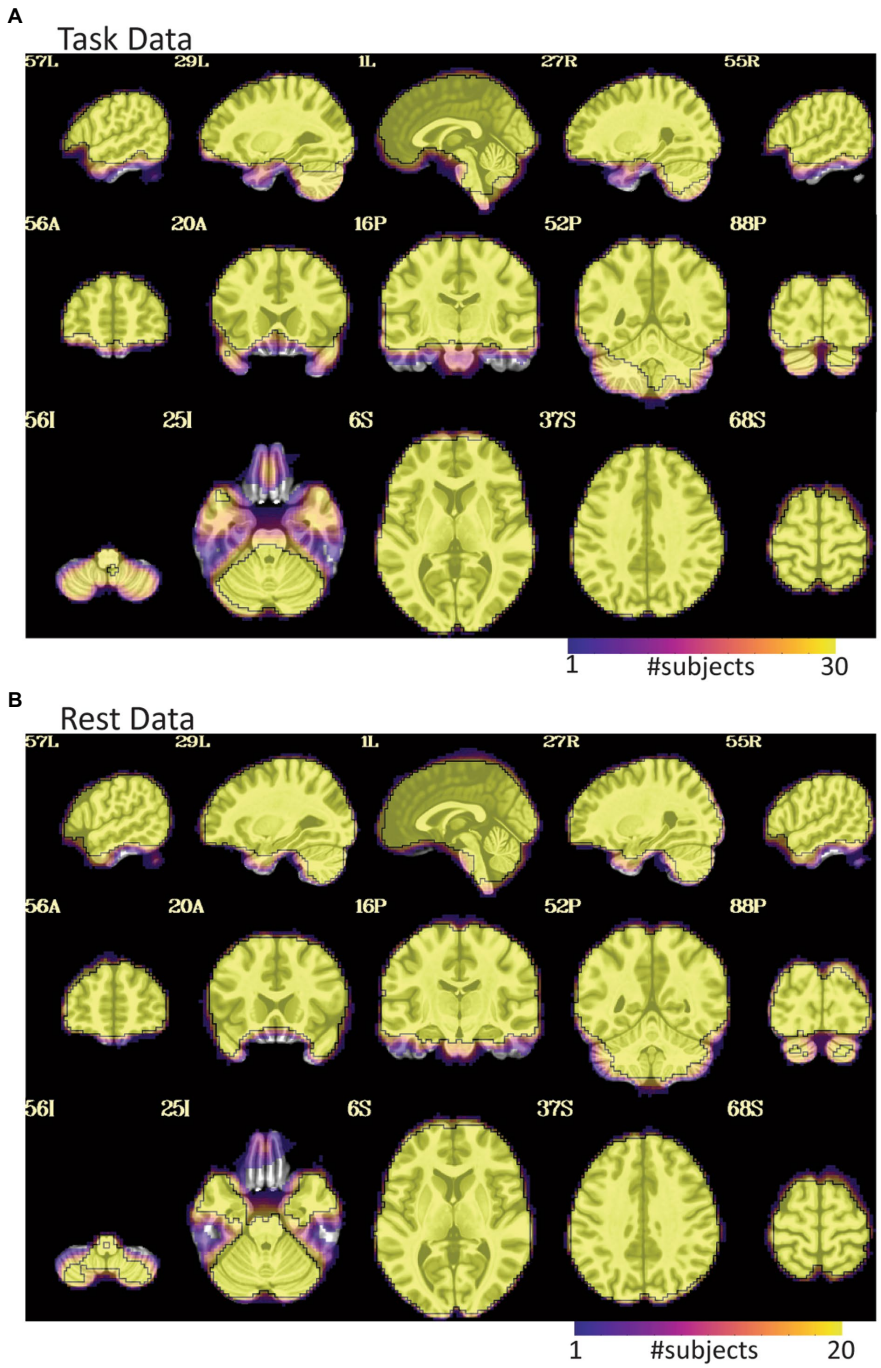
EPI coverage maps in MNI space for **(A)** task and **(B)** rest data sets. More yellow indicates that more subjects retained usable data for a given voxel. More purple indicates voxels where fewer subjects have usable data. The black outline surrounds voxels where all subjects have useable data. While both datasets show dropout in orbitofrontal and inferior temporal areas, the dropout is less consistent and more pervasive in the task data where much of the temporal lobe does not have usable data in a non-trivial fraction of subjects. The black line in **(A)** also highlights that not all subjects have cerebellar coverage.

**Figure 4:**
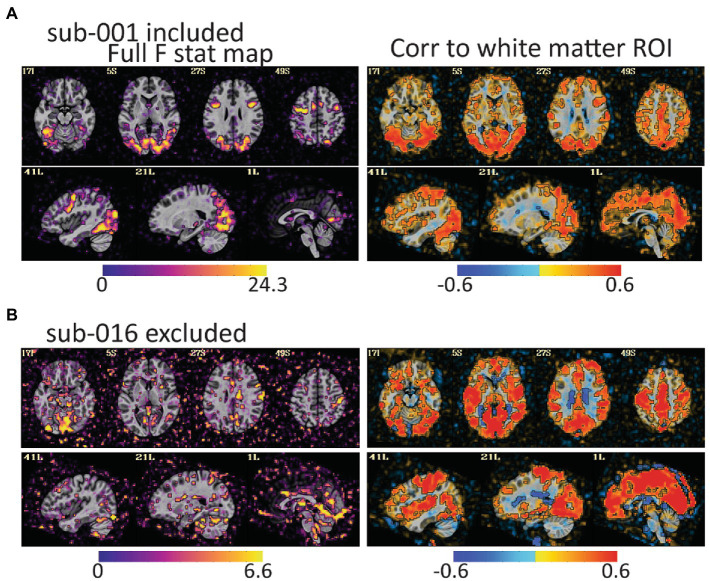
The full F stat map shows the decile of voxels with the highest *F* values for the full task GLM. The correlation to the white matter ROI shows voxels that correlated to white matter after the task design is regressed from the data. **(A)** For sub-001\u00B0F stat peaks are large and mostly in gray matter. **(B)** For sub-016, the F values are smaller, and the peaks are in lateral ventricles, CSF, and outside of the brain. The white matter correlation maps are harder to identify as clearly good or bad, but more pervasive correlations to gray matter as in (B) are an additional warning of a problem. Notably, both subjects have relatively little head motion (1.7% of volumes censored for sub-001 and 3.7% of volumes for sub-016) but AFNI also flagged sub-016 as having the task condition and not the control condition mildly correlated to motion. These maps provide evidence that task-correlated motion affected data quality.

**Figure 5:**
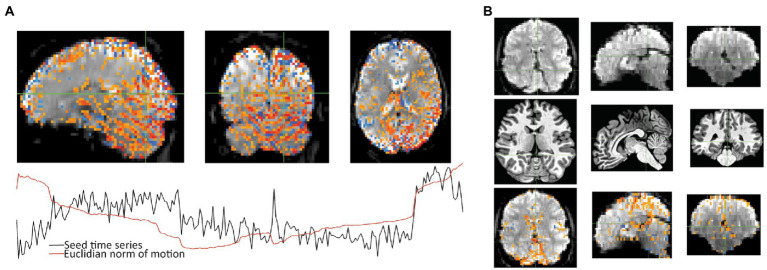
After seeing warnings due to “extent of local correlation” and “EPI variance lines” in AFNI’s automatic QC, *instacorr* was used to examine more closely. **(A)** For the correlation seed at the crosshair, Sub-018, shows an artifactual pattern of correlations (*p* < 0.001) across large portions of the posterior cortex and cerebellum. Time series shows that some of this follows several large jumps in motion. **(B)** For Sub-002, an unusually large hypointensity was noticed in the unprocessed EPI that was alarming during the initial review. Anatomical viewing of the same slices shows a slightly large superior cistern and 4th ventricle. Correlations to the cross hairs on the unpressed image (*p* < 0.001 with translucency below threshold) shows slightly larger correlations to CSF in the interhemispheric fissure. This observation will likely not cause problems for univariate statistical tests, but it could cause analysis issues if ROIs include this larger area of CSF that contains some internal correlations.

The task data contained numerous problems during the initial visual inspection process. Across the dataset, dropout and distortion were substantial in the unprocessed images. There were also very visible motion artifacts (e.g., Figures 1, 2). Four subjects were all automatically excluded because more than 20% of volumes exceeded motion and outlier censoring thresholds. Most subjects showed substantial dropout in the temporal lobe and some showed cerebellar dropout (Figure 3A). Several subjects showed atypically high correlations between a white matter ROI and gray matter voxels and areas of highest activation to the full F test for the task outside of the brain or in CSF (Figure 4). Based on EPI variance line warnings, visual inspection with *instacorr* identified several subjects with non-trivial artifacts (Figure 5A). Additionally, multiple subjects showed mild to moderate correlations between the task and control condition timing, which reduces that statistical power to independently estimate effect sizes for the two conditions. Since we did not create the study design, we did not exclude any participants solely because of this correlation. In total, 14 subjects were marked for inclusion, 12 were marked for exclusion, and 4 were marked unsure out of a 30-subject data set. An overview of our findings across both datasets are shown in Table 1.

**Table 1:** QC Classifications for all subjects.

Subject ID	Status	Notes
sub-001	Include	Dropout in temporal lobe
sub-002	Include	Dropout in temporal lobe
sub-003	Unsure	Dropout in temporal lobe; larger corr between white matter ROI & gray matter; 10.3% vols censored; instacorr showing potentially serious motion artifacts
sub-004	Include	Dropout in temporal lobe
sub-005	Unsure	Dropout in temporal lobe; larger corr between WM & GM; Instacorr shows several widespread artifacts, possibly respiratory
sub-006	Include	Dropout in temporal lobe; larger corr between WM & GM
sub-007	Include	Dropout in temporal lobe
sub-008	Include	Dropout in temporal lobe
sub-009	Exclude	35% vols censored; very large corr between WM & GM; activation hotspots outside of brain
sub-010	Include	Dropout in temporal lobe; larger corr between WM & GM
sub-011	Include	Dropout in temporal lobe
sub-012	Exclude	*Instacorr* showing some artifacts; 12.8% vols censored; Full F stat map hotspots outside of brain and speckled inside brain; Dropout in temporal lobe
sub-013	Exclude	10.3% vols censored, task vols more censored than control; Full F stat map hotspots outside of brain and speckled inside brain; *instacorr* showing some artifacts; Dropout in temporal lobe
sub-014	Include	Dropout in temporal lobe; larger corr between WM & GM
sub-015	Include	Larger corr between WM & GM
sub-016	Exclude	Dropout and distortion in temporal and frontal lobes affecting alignment; activation hotspots in CSF; task correlation to motion
sub-017	Exclude	40% vols censored; Dropout in temporal lobe
sub-018	Exclude	*Instacorr* showed nontrivial MRI artifact correlations
sub-019	Include	Dropout in temporal lobe; larger corr between WM & GM
sub-020	Include	Dropout in temporal lobe
sub-021	Include	12.4% vols censored; Dropout in temporal lobel and cerebellum
sub-022	Exclude	*Instacorr* showed nontrivial MRI artifact correlations; 17.8% vols censored; Full F stat map speckled inside brain; Dropout in temporal lobe
sub-023	Exclude	Hotspots of activity outside of brain and little robust in-brain hotspots; very large corr between WM & GM; radial corr map shows probably artifacts; 14.9% vols censored; Dropout in temporal lobe
sub-024	Exclude	33.5% vols censored
sub-025	Exclude	15.3% vols censored; task-correlated motion; more motion censoring in task vs. control; very large corr between WM & GM
sub-026	Unsure	19.4% vols censored; larger corr between WM & GM; Dropout in temporal lobe; slightly more censored vols in task vs. control
sub-027	Exclude	19.4% vols censored; Hotspots of activity outside of brain and little robust in-brain hotspots; very larger corr between WM & GM; Dropout in temporal lobe
sub-028	Include	7.9% vols censored
sub-029	Exclude	20.2% vols censored
sub-030	Unsure	*Instacorr* and local corr maps showed localized artfacts that might require exclusion depending on areas of research interest
sub-101	Exclude	Likely Left/right flip; 20.5% vols censored
sub-102	Include	5.8% vols censored
sub-103	Include	2.6% vols censored; *instacorr* correlations not great, but nothing clearly exclusionary
sub-104	Include	16% vols censored; *instacorr* correlations not great, but nothing clearly exclusionary
sub-105	Include	11.5% vols censored; *instacorr* correlations not great, but nothing clearly exclusionary
sub-106	Exclude	13.5% vols censored; Very large global correlations to seeds
sub-107	Include	19.2% vols censored
sub-108	Include	4.5% vols censored
sub-109	Include	3.8% vols censored
sub-110	Include	4.5% vols censored
sub-111	Exclude	7.7% vols censored; Very large global correlations to seeds
sub-112	Include	6.4% vols censored
sub-113	Include	0.6% vols censored
sub-114	Exclude	4.7% vols censored; Very large global correlations or anti-correlations to seeds
sub-115	Exclude	Likely left/right flip
sub-116	Exclude	Neither left–left nor left/right flip is great. With close inspection, unclear if anatomical is same brain as EPI
sub-117	Include	1.9% vols censored
sub-118	Exclude	30.1% vols censored
sub-119	Include	10.9% vols censored
sub-120	Include	1.3% vols censored; *instacorr* correlations not great, but nothing clearly exclusionary

Task subjects are 001–030, rest subjects are 101–120. Notes explain why a subject was excluded or unsure or highlight something worth continued monitoring in included subjects.

**Figure 6:**
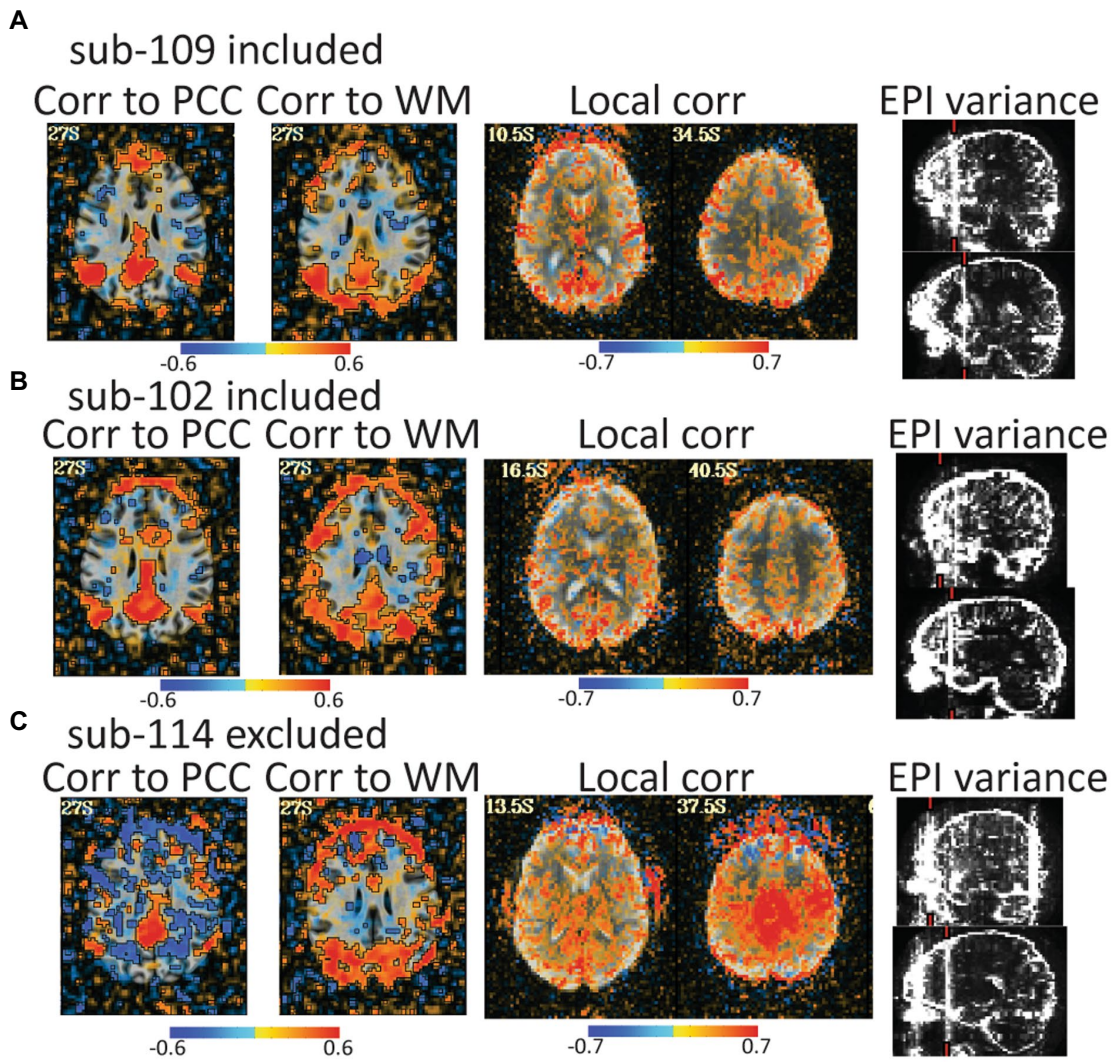
Automated QC image from 3 rest data study subjects with low head motion (only 4%–6% of volumes censored). An atlas-based posterior cingulate (PCC) ROI is calculated and the correlation maps (r values), should highlight some default mode network (DMN) connections. Too much correlation between a white matter (WM) ROI and gray matter can be concerning. Local correlations are the correlations of each voxel to surrounding voxels in a 2 cm sphere and can highlight scanner artifacts. EPI variance line warnings highlight lines of high variance that might be artifacts. **(A)** sub-109 has a plausible DMN from the PCC seed, no excessive correlations to white matter, no non-anatomical local correlations, and the variance warnings were checked with *instacorr* and did not show pervasive issues after preprocessing. **(B)** sub-102 was typical for these data. The DMN is present, but not as clean, there are more WM correlations in and out of the brain, and EPI variance warnings showed some issues with *instacorr*, but not enough to reject. If typical subjects in this dataset were cleaner, we might have rejected sub-102. **(C)** sub-114 is a clear rejection with non-anatomical anticorrelations to the PCC, large artifacts in WM correlations, a large local correlation, and EPI variance warnings paired with concerning artifacts visible with *instacorr*.

**Figure 7:**
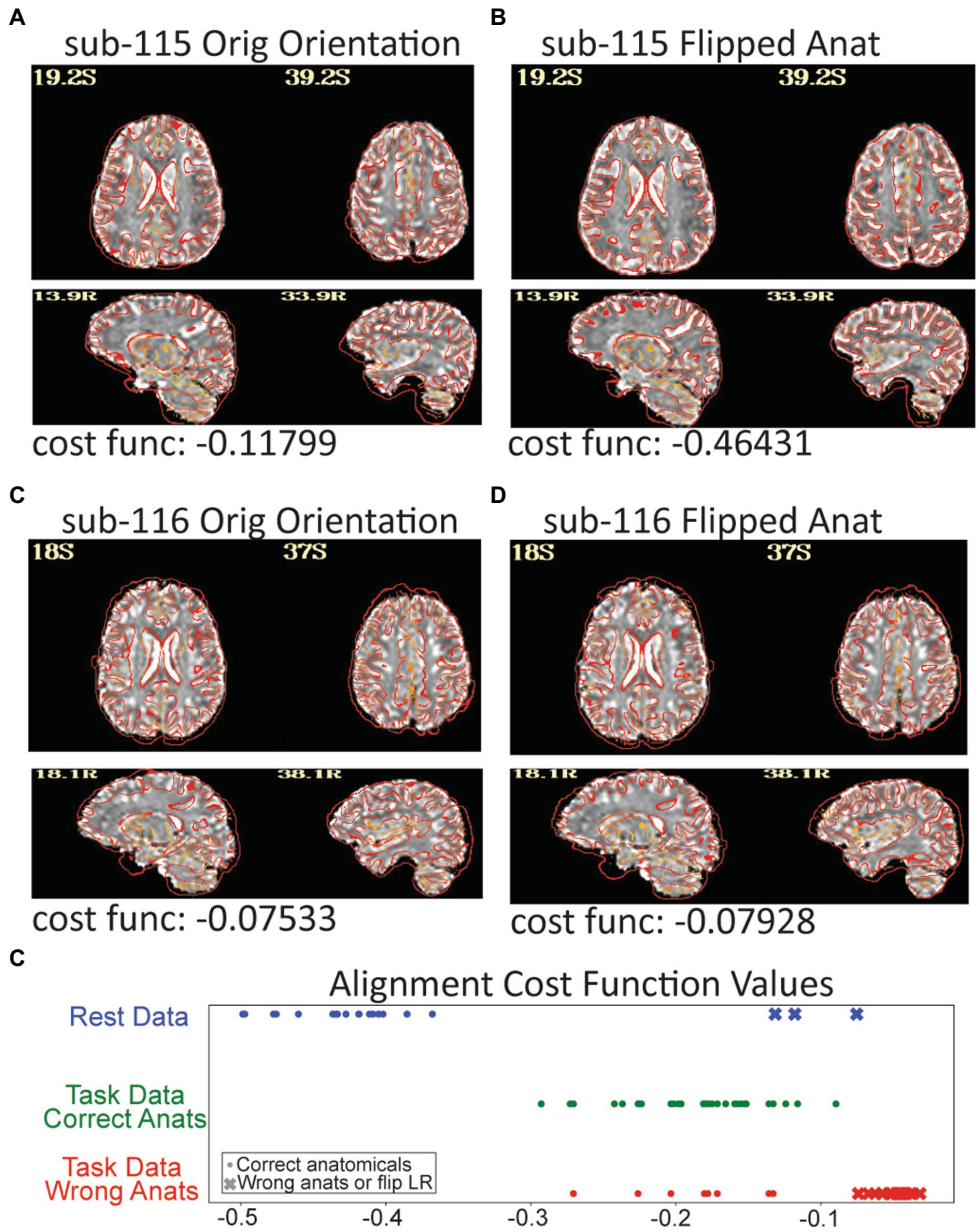
Three subjects in the resting data triggered a left–right flip warning which happens when the cost function for anatomical to EPI alignment finds a better local minimum after flipping the anatomical. The grayscale EPI image used for alignment is shown with the edges of the aligned anatomicals. **(A)** The original alignment for sub-115 looks ok, but **(B)** shows the alignment for sub-115 with the anatomical image flipped and the gyral edges are clearly better matched. Sub-115 generated a “severe” left–right flip warning. Sub-116 does not have a great alignment for the original **(C)** or flipped **(D)** anatomical and generated a “medium” left–right flip warning. Since neither fits well, sub-116 may have been shared with the wrong anatomical image. **(E)** The cost function minimums for the successful alignments in the rest dataset were − 0.36 to −0.5 while the 3 flipped alignments were more than −0.13. Similarly, when the task data were unintentionally aligned to the wrong anatomicals, the cost functions were much higher. While cost functions are relative measures, the values may be useable as an intra-study alignment QC measure.

For the rest data, more of the brain was consistently covered (Figure 3B). Two subjects were automatically excluded because more than 20% of volumes exceeded motion and outlier censoring thresholds. Areas of general concern in the rest data included correlations between gray matter and a white matter ROI, poor correlations to expected networks from ROIs like the posterior cingulate, and EPI variance line warnings followed by *instacorr* inspection of artifacts. In these data, *instacorr* often showed issues related to EPI variance warnings in the unprocessed EPIs, but when censored volumes were removed by processing, *instacorr*-observable artifacts were reduced, and the remaining data were usable. The threshold between inclusion and exclusion based on these criteria was subjective, and the decision to exclude was typically based on several borderline reasons for concern, such as more than 10% of volumes censored and signs of artifacts in the data. We likely would have excluded more subjects if other subjects with this study were less noisy (Figure 6). Two subjects were excluded because the left and right sides of the brain were likely flipped between the anatomical and EPI data and an additional subject looked like the anatomical volume was from a different brain than the EPI (Figures 7A–D). Given 3 participants showed an EPI and anatomical mismatch, there is a risk of an underlying issue with file naming and organization in these data. If we were using these data as part of a study, we would try to identify the origin of the flipping to confirm the scope of the problem and possibly identify the true left vs. right so that these participants would not need to be excluded. In total, 13 subjects were marked for inclusion and 7 for exclusion out of a 20-subject data set.

## 5. Discussion

We outlined priorities for QC of fMRI studies and then demonstrated them on two datasets. While priorities are best organized around conceptual goals, QC steps are ordered by when potentially serious problems are noticed. For the exemplar data, high motion, non-trivial distortion or dropout, and warnings signs for artifacts were rapidly apparent and dominated our focus. We highlight TSNR and several other measures as important QC metrics in our priorities, but we did not highlight them in practice. This is because some data did have low TSNR and artifacts that were clearly visible in TSNR maps, but these were in runs that were already rejected for other reasons. For these data, TSNR measures might have improved understanding of the effect of motion artifacts, but TSNR did not add value to decisions of what to include or exclude. In other datasets, TSNR has been the first place where something problematic is noticed.

This emphasizes a critical point of QC protocols. Datasets can have unique quirks, and the most useful QC checks for fMRI data are not universal across all studies. We’ve interacted with researchers who had a bad experience with head motion in a study and prioritized checks for head motion above all else. In fact, when the Organization for Human Brain Mapping put together a consensus statement on results reporting, it included a general recommendation to document QC measures, but only specified motion and incidental findings for fMRI data (Nichols et al., 2017). Reporting on alignment quality, MRI artifacts, degrees of freedom available, and consistency of the imaging field of view were not mentioned. For QC to become an intrinsic part of data acquisition, processing, and sharing, guidelines should be updated to include at least these valuable QC metrics.

A good QC process is designed to identify and address issues as soon as possible. The shared task data had many problems that were not addressable by the stage we received them. With the goal of improving the quality of shared data, we want to highlight QC steps that could have helped avoid collecting a dataset with such problems. Some problems, like the artifacts from extreme motion depicted in Figure 1, should have been observable during data acquisition. Real-time motion tracking, would identify high motion runs during scanning and potentially create an opportunity for additional acquisitions. Additional real-time monitoring of peripheral data, like eye tracking, behavioral responses, or cardiac and respiratory traces would identify drifts in consciousness or attention to the task. Once data are collected, rapidly running some subject-level analyses may identify correctable problems. For example, many of the acquisition issues in the rest data that might cause the spatio-temporal artifacts we saw would have been visible early in collection and might have been fixable through changes in acquisition. We reiterate that it is imperative to run analyses as early as possible to avoid acquiring large amounts of data with problems that do not arise until the study is analyzed months or years after acquisition began.

Between the original and revised submission of this manuscript, we noticed a serious error in our processed data that we missed even while using a detailed QC protocol. For a subset of task subjects, the skull stripped anatomical volumes were mis-labeled and we aligned fMRI data to the wrong anatomicals. This created an unintentionally good case-study on the limits of QC and how we could have caught this error earlier. We introduced this work by stating the purpose of QC is to identify whether data is of sufficient quality to be used for its intended purpose. In this case, we observed bad EPI to anatomical alignments, and wrote that the data would be not usable for their intended purpose until alignment was fixed. If we planned to use these data for a larger study, we would have tried to fix the alignment, but for this demonstration, we ended by noting the alignment issues. This occurrence highlights how human interpretation is a fundamental part of QC and understanding why data are low quality is sometimes more important than merely identifying low quality data. Even while emphasizing the importance of human interpretation, we leaned too heavily on an automated summary image to reject an alignment. This is a critical point since, as study sample sizes increase and data rejection is automated and not followed up by human interpretation, the more likely usable data will be automatically rejected and systematic issues underlying data rejection will be overlooked.

Automated measures combined with human interaction and judgement were essential to the QC process. While automated measures such as correlations to a white matter ROI, statistical result maps, and line variance warnings mandated closer attention, it was direct inspection of volumes and time series, including with using *instacorr*, that became essential for identifying wide-spread issues that warranted data exclusion. Our initial error with mismatching anatomicals and EPIs also highlights the importance and limits of automated QC for registration. The alignment measures showed bad alignments, but not why. For several subjects, the mismatched volumes were subtle even with a close inspection. AFNI’s warning for left–right flips is an example where automation can highlight a serious alignment problem that is also subtle. More innovation in automated metrics to assess alignment quality, such as the demonstrated left/right flipping test, is needed. For example, a *post hoc* analysis of our mismatched processing showed that while the cost functions used for alignment are sensitive to the precise contrasts of the EPI and anatomical volumes, since the anatomicals and EPI images had similar contrasts across the dataset, the cost function values for the mismatched fits were clearly higher than the good fits in comparison to other subjects in each dataset (Figure 7E). This is a potential new automated metric that could flag concerning alignments for follow-up by human inspection.

At many points in this project, it was clear that hands-on training was essential. The two authors who conducted most of the visual inspect of results have been working with fMRI data for slightly more than a year. Though the more experienced authors gave consistent instructions, it was impossible to give them comprehensive written instructions that covered the range of issues they observed solely within these datasets. For example, there were several cases where anomalies in images, like a line of CSF that was unusually visible in a single slice caused serious concerns during the initial review, but expert feedback showed it was not a serious problem (Figure 5B). Improving the training of novice neuroimagers was an interactive and iterative process, where they presented concerning observations and the more experienced neuroimagers helped them understand what issues were or were not actual concerns. Over time, they were able to more independently make appropriate QC judgements. Therefore, such training needs to go beyond a lecture and involve mentored examination of actual datasets.

We have endeavored to provide some points of discussion when devising ways to train people in QC and provide a stable framework for creating a process tailored to individual researchers’ needs. Teaching best practices for quality control is far beyond the scope of a single manuscript. Since we focus on QC, rather than what to do after QC, we do not substantively discuss MRI artifacts nor ways to reduce certain artifacts through changes in acquisition or analysis. There are existing reviews on fMRI noise and noise reduction (Liu, 2016; Caballero-Gaudes and Reynolds, 2017), but we are not aware of any published reviews or even book chapters that specifically focus on MRI artifacts for fMRI. While recorded lectures and blog posts cover MRI artifacts, learning to understand and interpret fMRI artifacts remains heavily dependent on hands-on training.

Automation remains essential to QC. Appropriate use of automation can be a very important part of both analysis and quality control when paired with human interpretation and rigorous inspection. When steps are properly automated, human induced errors can be reduced, resulting in more consistent and reproducible results across subjects or analyses. Automated pipelines are also more likely to be neatly organized and understandable, with notes integrated into the scripts that run them rather than scattered across an entire project. This can drastically ease the burden of finding important data or tables to inspect. For the QC metrics demonstrated here, head motion, temporal outlier detection, DOF counts and accompanying censoring and warnings were automatic and appeared robust. Flagging of left–right flipping, while only partially automated, proved invaluable as it is a very difficult problem to spot. Additionally, having a report which organizes much of the relevant information in one place to systematically review, saved many personnel hours during the data review process and made it possible for the human review to efficiently focus on actual issues.

We used and benefitted from many automated QC measures that are now built-in defaults when AFNI’s *afni_proc.py* command is run. Automation is a work in progress and each tool has strengths and weaknesses. We note some places where AFNI’s automation can improve under the assumption that these may benefit other tools as well. In particular, connections between reports and the underlying data that generated them could be improved so that it could be easy to quickly navigate to from a concerning image, such as an image of a few slices with questionable alignment, to explore the full alignment in more depth. Another gap in AFNI’s automated measures is that there are few automated summaries of QC measures across participants.

The publication describing *MRIQC* tools discusses potential inconsistencies by basing too many decisions on human judgements and recommends a push toward more automated measures (Esteban et al., 2017). While we agree automated measures are essential and they acknowledge human judgement is still important, we think there can be dangers from over automation or excessive trust in automated thresholds for QC metrics. Automated measures can suffer biases of omission. For example, the lack of automated measures for alignment quality is paired with the lack of a field-wide discussion on the noise and reproducibility issues due to sub-optimal alignments. Automated measures that reject data without human interpretation can also mask underlying and solvable issues.

We believe it is imperative to continue discussing QC priorities, processes, standards, and tools. Moreover, discussions of reproducibility and reliability of fMRI data need to go beyond concerns over head motion and precise yet arbitrary statistical thresholds. Focusing just on one QC concern, like head motion, is like a building inspector looking for signs of water damage. Water damage can be a serious issue and expertise is required to know how to look for such damage, but there is a risk to over-focusing on water damage and missing signs that the floor is about to collapse. Good quality control requires a more comprehensive assessment. The neuroimaging community can do more to understand the full range of problems that exist in data today, so that we can get better at identifying and documenting problems. Particularly as data sharing becomes the norm, the more we can do to improve QC processes today, the more likely our current data will still be useful for future research.

We have demonstrated a typical QC process for our research group. We have likely missed some data quality problems that other researchers may catch because processes vary and are often tailored to different research approaches. This is one of the reasons we highlight the importance of the underlying scientific questions and context for good QC. We hope more researchers will share their QC protocols, so that a wide array of approaches can be compared and used to improve the next generation of QC tools and processes.

## 6. Conclusion

Good data quality is essential for reproducible science. Quality control processes help validate data quality and ensure data are suitable to address experimental questions. Timely QC steps during the early stages of a study can improve data quality and save resources by identifying changes to acquisitions or analyses that can address problems that arise during QC. QC is an ongoing process that does not end after the early stages of a study. Shared data are not inherently quality-checked data, and even shared data that includes a documented QC process and output may not be sufficient since priorities for quality checks can be study context-dependent.

A good QC process should be integrated into study planning. While automation should be used wherever possible, human observations and interpretations are critical. Much discussion of QC focuses on the binarized decision of whether to keep or exclude data, but we find that a key element of QC is to identify potentially correctable issues. Particularly, as fMRI studies increase in size or aggregate multiple datasets, good QC processes will require planning that includes decisions on what can be automated and what will require peoples’ time.

Much public discussion about reproducible neuroimaging has focused on appropriate sample sizes, statistical tools, and thresholds. We posit that normalizing timely and rigorous QC is an equal if not more important step our field can take to improve reproducibility. While we present a framework for thinking about fMRI QC along with a demonstration of one existing QC pipeline on a couple of shared datasets, this is far from sufficient. Quality control priorities and methods deserve more attention, discussion, and innovation from the neuroimaging community.

## Data availability statement

Publicly available datasets were analyzed in this study. This data can be found at: https://osf.io/qaesm/.

## Ethics statement

The studies involving human participants were reviewed as part of this special issue. The editors selected openly available ethics committee approved datasets, which we analyzed without knowing the source of the data. The patients/participants provided their written informed consent to participate in this study.

## Author contributions

JT, DH, JG-C, and PB contributed to overall study design. JT wrote the processing scripts and guidelines for running and checking the exemplar data. MH, MS, and DH processed most data and did the quality checks under the direct supervision of JT with active support and guidance from DH and JG-C. DH and JT wrote the manuscript with some figures by MS and MH. All authors gave feedback on the manuscript. All authors contributed to the article and approved the submitted version.

## Funding

This research was supported by the Intramural Research Program of the NIMH under grant number ZIAMH002783.

## Conflict of interest

The authors declare that the research was conducted in the absence of any commercial or financial relationships that could be construed as a potential conflict of interest.

## Publisher’s note

All claims expressed in this article are solely those of the authors and do not necessarily represent those of their affiliated organizations, or those of the publisher, the editors and the reviewers. Any product that may be evaluated in this article, or claim that may be made by its manufacturer, is not guaranteed or endorsed by the publisher.

## Footnotes

^1^QC Project main page: https://www.frontiersin.org/research-topics/33922/demonstrating-quality-control-qc-procedures-in-fmri

^2^ https://github.com/nimh-sfim/SFIM_Frontiers_Neuroimaging_QC_Project
