# Supplementary material for: The art and science of using quality control to understand and improve fMRI data
Source: Front Neurosci. 2023 Apr 6;17:1100544. doi: 10.3389/fnins.2023.1100544 (PMC10117661; doi:10.3389/fnins.2023.1100544)
Supplement: Supplementary file 1 [file Table_1.docx]

Teves JB, Gonzalez-Castillo J, Holness M, Spurney M, Bandettini PA and Handwerker DA (2023) The art and science of using quality control to understand and improve fMRI data. *Front. Neurosci.* 17:1100544. doi: 10.3389/fnins.2023.1100544

**Appendix**

The following is a list of priorities to consider as part of a Quality Control Protocol. It is designed to be summary of the key points in this manuscript that can guide the creation of a study-specific QC protocol. While these priorities are independent of processing software, an ^*^ denotes information that is collected within *afni_proc.py*’s automatically generated QC report or from logs explaining a script failure. QC measures from other AFNI functions are listed. Measures that are demonstrated in our github repository but aren’t automatically generated by AFNI are denoted with a ^+^.

| **Priority** | **Context & Examples** |
| --- | --- |
| **General** | |
| Which voxels have usable data? | Voxel-wise data quality & coverage^*^ |
| Are locations of voxels accurately defined? | Distortion & alignment to anatomy & templates^*^ |
| Define context | Scientific questions & study priorities affect what is or is not good quality data |
| **During study planning** | |
| QC measures to support study goals | Particularly for study-specific QC priorities, this is a good time to seek expert advice |
| Operation procedures to decrease acquisition errors | Good procedures are critical for making sure data are accessible and consistently documented |
| Additional measures to collect | Experimenter notes, behavior logs, respiratory & cardiac traces |
| Organization & sharing QC measures | Inaccessible information is not useful |
| Piloting acquisition & processing | Evaluate and improve a QC protocol as part of study piloting |
| **During Acquisition** | |
| Real-time monitoring of severe image distortions, head motion, task non-compliance | Observing problems during acquisition can give time to recollect data or fix problems for the current or future scans |
| Monitor peripheral measures | Respiration, cardiac, eye tracking |
| **Soon after acquisition or download** | |
| Expected data are all present and properly documented | Missing, duplicated, or corrupted files, incomplete runs.^*^ For MRI data, behavioral logs, and peripheral measurements |
| Data consistency & documented parameters match data | Consistent MRI field of view, contrast, orientation, number of runs, & run lengths match documentation^*+^ |
| Documentation on QC during acquisition or pre-sharing exists | No documentation means there are undocumented problems |
| Data plausibly useful for study goals | Regions of interest should have full coverage. No substantive temporal artifacts that affect connectivity measures |
| Atypical brain structures, acquisition artifacts, drop out, and distortion | May still be fine, but might require altered processing. AFNI’s *instacorr* can be useful for assessment |
| **During and after processing** | |
| Scripts ran properly | Expected logs, QC metrics, & outputs created^*^ |
| Appropriate voxels retained or removed | Voxels with good SNR in brain are within mask and voxels outside of brain are removed.^*^ |
| Voxels lost to dropout or field of view | Check that similar voxels are retained across the population^+^ |
| Consistent measures of temporal signal-to-noise and intrinsic spatial smoothness across population | Sessions with non-trivially lower TSNR or different smoothness can be a warning sign of other problems^*^ |
| Automatically removed data | Number of censored volumed and DOF lost from noise regression, temporal filtering, & censoring^*^ |
| Artifacts like ghosting, phase wrapping, or leakage | *Instacorr* is useful for checking if the temporal signal from an article is folding over into other brain regions |
| Partially-thresholded activation maps | Are areas with the largest model fits in anatomically plausible patterns inside the brain?^*^ |
| Task correlated head motion or breathing | Not commonly checked and can bias results.^*^ (AFNI automatically checks motion, but not breathing.) |
| Skull properly masked for anatomical & functional data | Can cause problems with alignment. Part of report from AFNI’s *SSwarper* |
| Intensity inhomogeneity | Brighter signal on the surface can be expected, but can cause problems with masking and alignment^*^ |
| Good anatomical to functional alignment & alignment across days/runs | Can be a serious hidden problem if one just looks at group maps.^+^ |
| Left & right hemispheres flipped between anatomical & functional data | More common than it should be & requires excluding data unless the true left/right can be determined^*^ |
| Good anatomical to anatomical alignment across participants | Often correctable and causes problems if not corrected^+^ |
| Group coverage across population | A summation of aligned functional masks highlights brain areas missing in part of the population^+^ |
| Processed peripheral data are good | Plausible behavioral timing files, good peak detection in respiratory & cardiac traces |
